# Unveiling the novel role of circadian rhythms in sepsis and septic shock: unexplored implications for chronotherapy

**DOI:** 10.3389/fendo.2025.1508848

**Published:** 2025-02-04

**Authors:** Stewart D. Ramsay, Marni A. Nenke, Emily J. Meyer, David J. Torpy, Richard L. Young

**Affiliations:** ^1^ Intestinal Nutrient Sensing Group, The University of Adelaide, Adelaide, SA, Australia; ^2^ Adelaide Medical School, The University of Adelaide, Adelaide, SA, Australia; ^3^ Lifelong Health, South Australian Health and Medical Research Institute (SAHMRI), Adelaide, SA, Australia; ^4^ Endocrine and Metabolic Unit, Royal Adelaide Hospital, Adelaide, SA, Australia; ^5^ Centre of Research Excellence in Translating Nutritional Science to Good Health, The University of Adelaide, Adelaide, SA, Australia

**Keywords:** septic shock, circadian rhythm, chronotherapy, chronopharmacotherapy, intensive care unit

## Abstract

Circadian rhythms are critical to coordinating body processes to external environmental cues, such as light and feeding, to ensure efficiency and maintain optimal health. These rhythms are controlled by ‘clock’ transcription factors, such as Clock, Bmal1, Per1/2, Cry1/2, and Rev-erbs, which are present in almost every tissue. In modern society, disruptions to normal circadian rhythms are increasingly prevalent due to extended lighting, shift work, and long-distance travel. These disruptions misalign external cues to body processes and contribute to diseases such as obesity and non-alcoholic fatty liver disease. They also exacerbate pre-existing health issues, such as depression and inflammatory bowel disease. The normal inflammatory response to acute infection displays remarkable circadian rhythmicity in humans with increased inflammatory activity during the normal night or rest period. Severe bloodborne infections, exemplified in sepsis and the progression to septic shock, can not only disrupt the circadian rhythmicity of inflammatory processes but can be exacerbated by circadian misalignment. Examples of circadian disruptions during sepsis and septic shock include alteration or loss of hormonal rhythms controlling blood pressure and inflammation, white blood cell counts, and cytokine secretions. These changes to circadian rhythms hinder sepsis and septic shock recovery and also increase mortality. Chronotherapy and chronopharmacotherapy are promising approaches to resynchronise circadian rhythms or leverage circadian rhythms to optimise medication efficacy, respectively, and hold much potential in the treatment of sepsis and septic shock. Despite knowledge of how circadian rhythms change in these grave conditions, very little research has been undertaken on the use of these therapies in support of sepsis management. This review details the circadian disruptions associated with sepsis and septic shock, the influence they have on morbidity and mortality, and the potential clinical benefits of circadian-modulating therapies.

## Introduction

Daily light and dark cycles entrain a set of oscillating ‘clock genes’ within the suprachiasmatic nucleus (SCN) of the hypothalamus, termed the ‘central clock’. In turn, this master central clock entrains and tunes rhythms in many body tissues to modulate cellular processes that optimise organ efficiency, anticipate the need for daily activity, and return to quiescence (1, 2).

**Figure 1:**
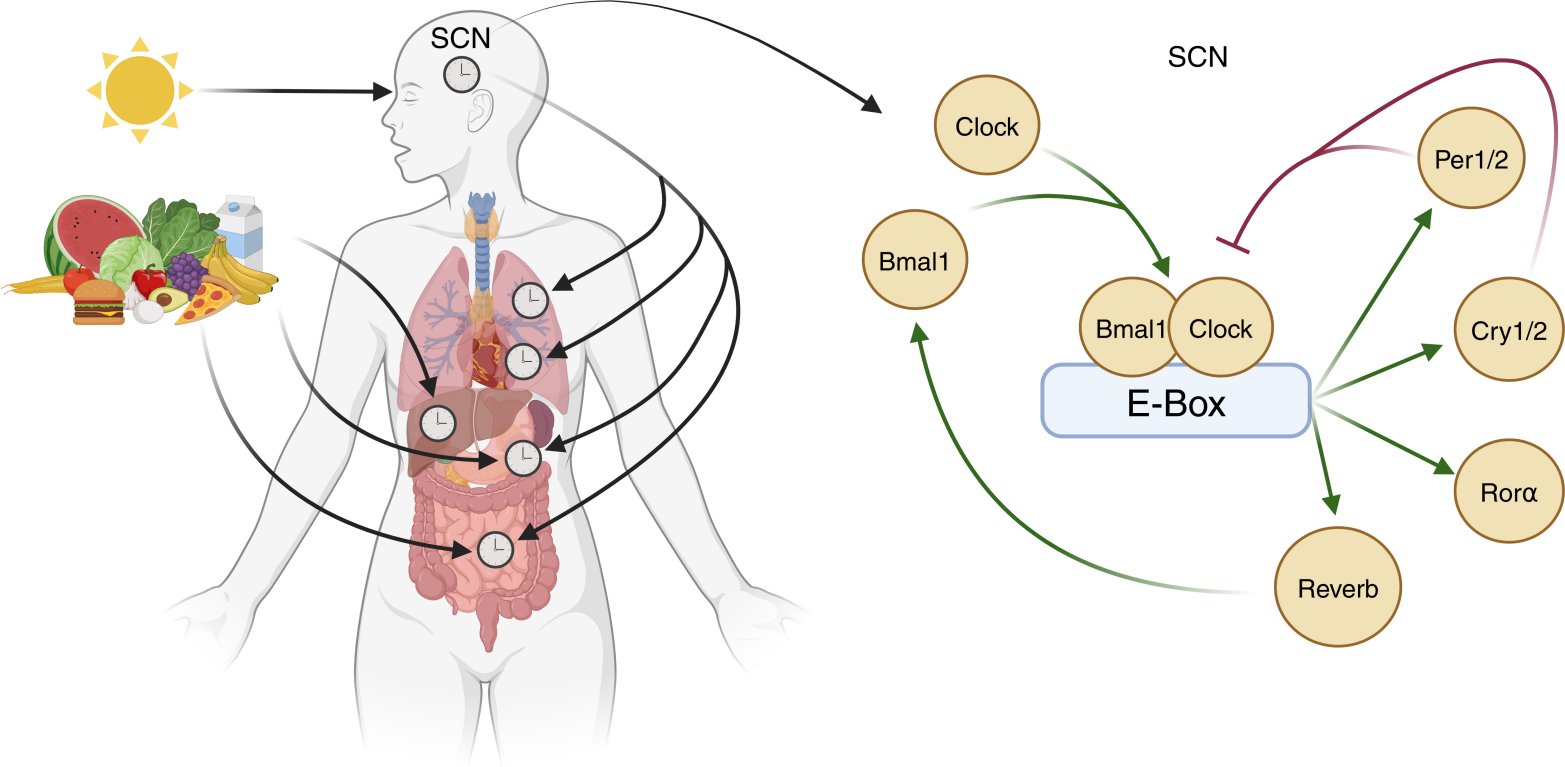
Entrainment of central and peripheral clocks. The central control of circadian rhythms occurs via light-dependent expression of Clock genes within the suprachiasmatic nucleus (SCN). Here, Clock and Bmal1 proteins dimerise and bind to the E-box of DNA to increase the expression of transcription factors Per1/2, Cry1/2, Rorα, and Rev-Erb. Rev-Erb increases Bmal1 expression whilst Per1/2 and Cry1/2 dimerise to inhibit Clock/Bmal1 activity. Circadian rhythms within tissue systems are also influenced by other factors, exemplified by meal timing effects in metabolic organs (image created in BioRender).

Circadian rhythms are influenced intricately by an array of external factors, notably light. In mammals, melanopsin-expressing photoreceptors detect the light-dark cycle and signal to the SCN via the retino-hypothalamic tract (1). In the SCN, glutamate receptor-driven Ca^2+^ influx evokes the expression of the gene products *Clock* and *Bmal1*. These dimerise and induce the expression of the transcription factors *Per1/2, Cry1/2, RORα*, and *Rev-Erb α/β*, all of which transcriptionally govern the cyclical expression of other genes (3). *Rev-Erb* also increases *Bmal1* expression, whilst *Per1/2* and *Cry1/2* dimerise to inhibit *Clock/Bmal1* activity (**Figure 1**). These feedback loops approximate a 24-hour cycle in mammals (4).

Non-light stimuli also entrain rhythms in peripheral organs (5). Nutrient intake, for example, is a strong peripheral cue that entrains the rhythm of clock genes in the gut and liver via insulin release, subsequently regulating *Per1/2* expression (6). Interestingly, inverting feeding times in mice results in a complete inversion of peripheral clocks in the gut relative to the SCN (7, 8), demonstrating the degree to which external factors influence tissue circadian clocks.

Disruptions to normal circadian rhythms, such as in shift work or jetlag, can have detrimental effects on health and exacerbate existing metabolic disorders. For example, chronic shift working conditions risk the development of obesity (9) and non-alcoholic fatty liver disease (10) and can exacerbate inflammatory bowel disease (11). The exacerbation of pre-existing disease by circadian disruptions is often overlooked in practice and not considered within treatment regimes. This is notable for in-hospital patients since hospital environments frequently extend periods of lighting, noise, and treatments into the night that disrupt circadian rhythms, particularly in intensive care settings (discussed in detail in Section 3).

Sepsis is characterised by a severe, systemic inflammatory response to a bloodborne pathogen, typically bacterial, while severe sepsis with concurrent hypotension requiring vasopressor therapy is termed septic shock. Sepsis is a leading cause of death worldwide, with a median 28-day mortality rate of 5–25%, compared to that of septic shock of 30-50% (12, 13). It has a global incidence of 49 million cases and accounts for 11 million deaths per year, representing 20% of all global deaths (14). Mortality, long-term rates of morbidity, and public health care costs for sepsis are rising due to advances in invasive surgery, implantable devices, and immunosuppression, with costs of $842 million per annum reported in Australia alone (15).

Standard care for septic shock comprises fluid resuscitation and the use of antimicrobials, with vasopressors (i.e., noradrenaline, epinephrine, dopamine) and ventilatory support as required. These protect the organs and extend the time for the patient to overcome the overwhelming inflammation; exogenous glucocorticoid (hydrocortisone, analogous to human cortisol) is used when these treatments no longer sustain blood pressure, offering vasopressor, metabolic, neurocognitive, and anti-inflammatory support (12, 13, 16–21). Multicentre studies show that exogenous hydrocortisone reduces the time to shock reversal; however, a 28- or 90-day mortality benefit is evident only in the most critically ill (16–18, 20, 21).

Substantial evidence advocates the safeguarding or preservation of circadian rhythms in best practice management of septic shock patients in-hospital, and during recovery. This review discusses the involvement of the circadian system in sepsis and septic shock, with a particular focus on the effects of the intensive care environment, endocrine rhythms, and critical components of the immune system.

## Clock genes in sepsis and septic shock

Serum transcript levels of the clock genes *Bmal1*, *Per2*, *Cry1/2*, and *Rev-Erb-α*/*β* show circadian rhythmicity in humans, with a peak at 1800 and nadir at 0800 for *Bmal1* and a peak at 2300–0200 and nadir at 1600–1800 for *Per2*, *Cry1/2*, and *Rev-Erb-α*/β. *Clock* transcripts, however, do not change (22–25). The rhythmicity of *Bmal1*, *Per1/2*, *Cry1*, *Rev-Erb-α*, and *Rev-Erb-β* in whole-blood is lost in patients with sepsis or septic shock, likely reflecting the changes in immune cells (22, 24, 25). Moreover, absolute levels of *Cry1* and *Rev-Erb-α*/*β* transcripts were reduced, and *Cry2* transcript was increased in the whole-blood of patients with septic shock (22, 24). A similar phenomenon occurs in the hippocampus and microglia of adult Sprague-Dawley male rats for *Clock*, *Bmal1*, *Per1/2*, and *Rev-Erb-α* in response to lipopolysaccharide (LPS)-induced sepsis (26). Together, these support the notion that septic shock disrupts both rhythmicity and absolute levels of circadian machinery.

It is known that C57BL/6 male mice with *Clock* deletion have increased survival from sepsis and reduced production of pro-inflammatory cytokines (27), indicating that the circadian system, or at least *Clock*, exerts detrimental effects in sepsis. However, evidence for a similar contribution of *Bmal1* disruption is equivocal. For example, upregulation of *Bmal1* in LPS-treated H9C2 cells increased cell viability by reducing ferroptosis and the accumulation of reactive oxygen species and lipid hyperoxides (28), whereas *Bmal1* depletion increased mortality in C57BL/6J male and female murine models of sepsis following caecal ligation and puncture (CLP) (29). Moreover, selective depletion of *Bmal1* in joint mesenchymal cells in a Col6a1-Bmal1^-/-^ mouse model of inflammatory arthritis enhanced cytokine production and augmented pro-inflammatory responses (30). In summary, despite being heterodimeric partners, a limited evidence base shows that *Bmal1* and *Clock* exert opposing anti-inflammatory and proinflammatory actions, respectively, in disease states.

## Circadian disruption in intensive care

Constant noise levels and extended lighting within intensive care units (ICUs) are significant disruptors of circadian rhythms (31–34). Indeed, patient disruptions due to therapy or monitoring in the ICU are the leading cause of circadian disturbances that alter sleeping patterns (34–37). However, changes in meal timing or form (e.g., enteral or parenteral) can also disrupt circadian rhythms, particularly those governing glucose homeostasis (38).

Sleep allows the body to rest, regenerate, and repair across four sleep stages: drowsiness (wake stage), light sleep (stage 1), deep sleep (stage 2), deepest non-rapid eye movement (REM) sleep (stage 3), and REM sleep (stage 4). Each stage corresponds with characteristic brain waves; the average human undergoes 4–5 cycles of these sleep stages per night (39). A decrease in overall sleep time (3–6 hours of sleep over 24 hours) and an increase in time spent in early sleep stages (stages 1 and 2) is reported in the ICU, with a significant decrease in time spent in latter sleep stages (stages 3 and 4) (35–37, 40, 41); however, one report suggests no changes to overall sleep duration (37). Interestingly, on-ward use of analgesics and sedatives also increases time spent in early sleep stages (36) and likely impairs sleep-aided recovery by limiting the latter sleep stages essential to the repair and growth of tissue. Thus, changes in sleep stage patterns and overall lower sleep quality in the ICU are likely to disrupt metabolic homeostasis and worsen defences against critical illness-induced inflammation.

The timing of food intake is an essential driver of tissue clocks in the liver and gastrointestinal tract and vital to preserving normal circadian rhythms. Intermittent parenteral feeding over 60 minutes every 4–6 hours preserves glucose homeostasis in healthy adults compared to continuous parenteral feeding; however, data on intermittent parenteral feeding in critically ill patients is lacking (42). In contrast, it is unsurprising that continuous parenteral feeding disrupts circadian rhythms and glucose homeostasis in ICU patients (38) and is known to disrupt the gut microbiome in a manner that is causally related to reduced tryptophan metabolites and insulin insensitivity in C57BL/6 male mice (43). It is unknown, however, whether intermittent parenteral feeding preserves glucose homeostasis and improves outcomes in ICU patients (44).

## Impact of sepsis and septic shock onset timing on mortality

It is unclear how the timing of sepsis onset relates to mortality in humans, given the difficulty pinpointing precise indicators of sepsis. While the time of septic shock onset is recorded within the ICU, a specific time-of-day onset association with mortality has not been interrogated. Rather, septic shock deaths are characterised as ‘early’ or ‘late’ when they occur prior to or after 3 days from ICU admission, respectively. It has been reported that the ICU admission time, i.e., day or night, does not affect overall ICU mortality, although weekend admission increases the risk of mortality (45, 46) and these studies were not specific to septic shock. Interestingly, an increased risk of mortality from sepsis and septic shock has been described in humans between 0200 and 0600 in a single report (47), supporting a time-of-day-dependency to mortality risk, requiring further investigation.

The relationship between the onset time of sepsis and mortality reported in nocturnal rodent models is relatively consistent for LPS-induced sepsis but inconsistent for polymicrobial sepsis (**Table 1**). In terms of Zeitgeber time (ZT, with ZT-0 defined as lights on and ZT-12 as lights off), LPS-induced sepsis onset during the light phase (ZT-0–11) led to increased morbidity and 70-100% mortality in Sprague-Dawley rats, C57BL/6 mice, and Bagg Albino mice in comparison to 20-50% mortality with dark-phase onset LPS-induced sepsis (ZT-12–19) (26, 48–51). However, the link between the timing of CLP, polymicrobial sepsis and mortality is less well-established and equivocal, with potential sex-specific outcomes (53) and a limited evidence base. For example, CLP performed in C57BL/6 female mice with a 1.5 cm caecal ligation in the dark phase (ZT-19) led to 100% mortality compared to 75% mortality in the light phase (ZT-7) (52). In contrast, CLP performed in C57BL/6 male mice with a 1 cm caecal ligation in the light phase (ZT-6–12) resulted in 85% mortality compared to 70% mortality in the dark phase (ZT-18–24) a difference that was lost in mice deplete in *Clock* (27) and related, in part, to sex-specific differences in clock gene control (54). However, a further study reported similar mortality after CLP was performed in C57BL/6 male mice in the light- (ZT-7) or dark-phase (ZT-19), despite the fact that mice that underwent dark-phase CLP reached 100% mortality faster at 60 hr, compared to light-phase CLP mice at 140 hr (55). The basis of these conflicting reports is unclear and requires deeper investigation.

**Table 1:** Impact of experimental sepsis timing on survival.

Induction	Species/sex/age	Timing	Mortality	Reference
LPS 5 mg/kg	Bagg Albino male, female mice, age not specified	4 hourly from ZT-0	▲ total at ZT-8/Light	(48)
LPS 20 mg/kg	C57BL/6 male mice 13–21-week-old	ZT-11:ZT-19 Light: Dark	▲ total at ZT-11/Light	(49)
LPS 25 mg/kg	Bmal^+/+^Lys-MCre male mice, age not specified	ZT-0:ZT-12 Light: Dark	▲ total at ZT-0/Light	(50)
LPS 0.1 mg/kg	Sprague-Dawley male rats, 8–12-week-old	ZT-6:ZT-16 Light: Dark	▲ total at ZT-6/Light	(26)
LPS	C57BL/6 male mice, 8-week-old	ZT-11:ZT-19 Light: Dark	▲ total at ZT-11/Light	(51)
CLP	C57BL/6 male mice, 6–8-week-old	ZT-7:ZT-19 Light: Dark	▲rate at ZT-19/Dark	(52)
CLP	C57BL/6 male mice, 8–12-week-old	ZT-6-12: ZT-18-24 Light: Dark	▲ total at ZT-12/Light	(27)
CLP	C57BL/6 female mice, 6–8-week-old	ZT-7:ZT-19 Light: Dark	▲ total at ZT-19/Dark	(52)

LPS, Lipopolysaccharide; CLP, Caecal Ligation and Puncture. Upward triangle represents an increase.

A more definitive study on sepsis timing and mortality was conducted in Sprague-Dawley male rats by Carlson and Chiu (56), who performed light-phase CLP (ZT-3–8) and then randomised rats postoperatively to a normal light cycle, a light-light cycle, or a dark-dark cycle. CLP rats exposed to the dark-dark cycle had significantly higher plasma corticosterone concentrations and one-week mortality (69%) compared to those in the light-light (38%) and normal light cycles (17%). These findings support the concept that in preclinical models, the onset timing of sepsis and loss of circadian cues during sepsis both strongly affect mortality in a potentially sex-specific manner.

## Impact of sepsis on endocrine systems

Sepsis triggers an immune hyper-response to infection and endocrine dysregulation, both of which contribute to organ dysfunction and damage. Activation of the hypothalamic-pituitary-adrenal (HPA) axis is the primary endogenous stress response to sepsis and triggers the production and release of vasopressors such as arginine-vasopressin (AVP), catecholamines such as epinephrine and norepinephrine, as well as cortisol in humans (57) and corticosterone in rodents (58). Major non-HPA hormones dysregulated during sepsis in humans and models of polymicrobial sepsis include melatonin and leptin. The circadian rhythms of these hormones are well-characterised in health., however, reports on the impact of sepsis on these rhythms are limited and equivocal and discussed in detail below and summarised in **Table 2**.

**Table 2:** Circadian changes to hormones during sepsis.

Hormone	Form	Health	Sepsis	Species/Sample	Reference
AVP	Clinical	–	No rhythm, initial increase followed by a sustained decrease	Human Plasma	(59)
Epinephrine	CLP	–	No rhythm, sustained increase	Sprague-Dawley male rat, Plasma	(60)
	LPS Endotoxemia	Peak 0800 Nadir 0400	No rhythm, sustained increase	Human (computer modelled)	(61)
Norepinephrine	CLP	–	No rhythm, sustained increase	Sprague-Dawley male rat, Plasma	(60)
Cortisol	Clinical	Peak 0500 Nadir 0100	No rhythm, sustained increase	Human Plasma	(62)
	Clinical	Peak 0600 Nadir 0200	Peak 1400 Nadir 2200	Human Plasma	(63)
	Clinical	–	No rhythm	Human Plasma	(64)
	Clinical	Peak 0800 Nadir 0000	No rhythm, sustained increase	Human Urine	(65)
Melatonin	Clinical	–	No rhythm	Human Plasma	(64)
	Clinical	Peak 0000 Nadir 0800	No rhythm	Human Urine	(65)
	Clinical	Peak 0200 Nadir 0800	No rhythm	Human Urine	(66)
	Clinical	Peak 0400 Nadir 0800	No rhythm	Human Plasma	(67)
	Clinical	Peak 0100 Nadir 0700	No rhythm	Human Urine	(68)
	Clinical	–	No rhythm	Human Urine	(69)
Leptin	Clinical	Peak 0100 Nadir 0900	No rhythm, sustained increase	Human Plasma	(62)
	Clinical	–	No rhythm, sustained increase	Human Plasma	70)

AVP, Arginine Vasopressin; CLP, Caecal ligation and puncture; LPS, Lipopolysaccharide.

### Arginine-vasopressin

AVP, also known as the anti-diuretic hormone, is produced primarily by SCN neurons and, to a lesser extent, paraventricular neurons and is released from the posterior pituitary (71). AVP binds to three G-protein-coupled receptors (vasopressin receptors (VR)): VR1a, VR1b, and VR2. AVP acts at VR1a on vascular smooth muscle cells to constrict blood vessels and increase systemic vascular resistance and is critical in blood pressure maintenance (72). AVP acts at VR1b to stimulate the release of adrenocorticotropic hormone (ACTH) from the anterior pituitary (73) and has an emerging role in regulating water permeability via intercellular crosstalk between renal VR1b and VR2 (74). AVP acts at renal VR2 to increase aquaporin-2 and promote the re-absorption of water and is essential in maintaining osmolarity (75).

Existing reports on the timing of AVP rhythms in the SCN and plasma are consistent in rodents. AVP concentrations and expression of the AVP1a receptor show a strong, inverse circadian rhythm in the SCN of 3-5-month-old C57BL/6 male and female mice; high plasma AVP concentrations at ZT-4 map to the nadir expression of the AVP1a receptor, while low AVP (ZT-16) maps to peak AVP1a receptor expression (76). These findings accord with peak AVP concentrations reported at ZT-2 and nadir at ZT-14 in the SCN of Wistar rats (sex unspecified) (77) and with peak bioluminescence of AVP at ZT-4 and nadir at ZT-15 in brain slices collected from C57BL/6 male mice (78).

Reports on AVP rhythms in human health are less consistent with peak AVP plasma levels reported at 0400 in adult males (aged 25 years), followed by an immediate decline to a nadir at 0800 (79). This contrasts reports in adolescents (14 years), where a prolonged AVP peak was reported from 0200 to 0800 and a nadir at 2200 (80). Moreover, absolute plasma AVP levels and AVP rhythmicity show age-dependent attenuation in older adults (70 years) (79).

Pathological settings, notably hypotension early in clinical and endotoxin-induced septic shock, increase AVP to supra-physiological levels (20- to 200-fold increase) in human, dog, and baboon plasma (81, 82), however, sepsis in C57BL/6 mice following CLP produced only a mild 4% increase in plasma AVP after 12 hours compared to sham mice (83). In humans, this increase is followed by a decline to sustained sub-physiological levels for up to 7 days (59). The initial increase of AVP in septic shock is due to the stimulation of its synthesis and release by the vagus nerve in response to hypotension (84, 85); however, the synthesis and production of nitric oxide, a negative regulator of vasopressin, is exacerbated during sepsis, contributing to the decline in AVP levels (86). Whether this decline in AVP is adaptive or maladaptive is not clear. However, it has been shown that plasma AVP levels increase in human septic shock non-survivors (87) which adds support to an adaptive change.

Clinical studies have reported circadian changes to AVP acrophase in enuresis (80), systemic atrophy (88) and hydrocephaly (89). To date, AVP levels have only been investigated in humans and modelled septic shock in cross-section, and longitudinal effects on AVP circadian rhythms are currently unknown, likely due to the persistently low level of AVP seen beyond early sepsis.

### Catecholamines

Epinephrine and norepinephrine are catecholamine neurotransmitters and hormones with distinct differences. Epinephrine is released mainly from the adrenal medulla during stress via the coordinated actions of the hypothalamic-pituitary-adrenal (HPA) axis and sympathetic nervous system. This occurs through neural connections between hypothalamic corticotropin-releasing hormones (CRH) neurons and brain stem autonomic nuclei A1, A2, and A6 (locus coeruleus) (90), and at the adrenal gland, where cortisol facilitates adrenomedullary phenyl ethanolamine N-methyltransferase enzyme activity for epinephrine synthesis (91). Epinephrine acts on cardiac and pulmonary β-adrenergic receptors to increase cardiac contractility and relax airway smooth muscle, respectively (92, 93). In contrast, norepinephrine is synthesised continuously in sympathetic neurons and targets α-adrenergic receptors in synergy with cortisol to constrict arterial vascular smooth muscle and increase blood pressure (94, 95).

Early studies reported that epinephrine and norepinephrine have similar circadian rhythmicity in the plasma of healthy adults, peaking shortly after waking at 0800 with a nadir at 0400 (96–99). However, the control of these rhythms differs: epinephrine rhythms are true circadian and persist without external cues, whereas norepinephrine rhythms do not meet the definition of a true circadian rhythm, and are lost in the absence of external cues (100, 101). α- and β-adrenergic receptors also display tissue-specific rhythmicity in binding capacity, with evidence of peak binding at ZT-20 and ZT-24 respectively in the brain of Sprague-Dawley male rats (102) and similar rhythms in the pineal gland of Sprague-Dawley male rats and Syrian male hamsters (103–105); there is limited knowledge on the rhythmicity of these receptors in other peripheral tissues.

Few studies have investigated the impact of sepsis on the circadian regulation of catecholamine levels due to their use as an exogenous therapy to sustain cardiac output and blood pressure in ICU standard care. Sepsis following CLP in Sprague-Dawley male rats led to a rapid (0.5 hours) increase in plasma epinephrine (10-fold) and norepinephrine (3-fold), that was sustained at 20 hours post-CLP, and in the absence of treatment, was coincident with a loss of the diurnal rhythm of plasma epinephrine and norepinephrine (60). Computer modelling using available data on the responses of TLR4, NF-κB, and heart rate to sepsis, also predicted an increase and loss of circadian rhythm of plasma epinephrine and norepinephrine in endotoxemia-induced sepsis in humans (61). Both catecholamines regulate cytokines responses to sepsis and other severe infections. Indeed, the infusion of epinephrine after LPS administration in humans reduces the levels of TNF-α and increases IL−10 levels in whole blood (106) adding support that catecholamines serve an anti-inflammatory role in sepsis.

### Cortisol (corticosterone in rodents)

Cortisol is a powerful immunomodulator produced by the adrenal glands in response to coordinated hypothalamic release of CRH, followed by CRH-dependent pituitary release of ACTH that converges on the adrenal gland to increase adrenocortical biosynthesis of cortisol (107). Cortisol prevents over-activation of the immune system to limit inflammatory damage to inflamed tissues (108), promotes gluconeogenesis and glycogenesis to increase blood glucose levels (109), and acts as a short-term vasopressor (110). Cortisol binds two receptors, the glucocorticoid (GR, *NR3C1*) and mineralocorticoid receptor (*NR3C2*), however, the GR is more relevant in responses to stress (111), sepsis and septic shock, and as such, the focus of this review. GR are present in almost all tissues and exists in two isoforms formed through alternate splicing of exon 9 (the inclusion of either exon 9α or 9β) to produce either GRα or GRβ. Alternate splicing for GRα produces a functional glucocorticoid binding C-terminal, while for GRβ, this produces a non-glucocorticoid binding C-terminal, that prevents it from mediating glucocorticoid effects (112). Upon cortisol binding, GRα translocates to the nucleus to bind glucocorticoid response elements acting as a transcription factor, or binds other transcription factors such as NF-κB, preventing them from acting on target genes (113). Cortisol-dependent changes in gene expression alter cellular functions to suppress T cell proliferation (114) and inhibit production of pro-inflammatory cytokines (107). Importantly, the GR is transcriptionally regulated by CLOCK and BMAL1 through acetylation, reducing GR-mediated transcriptional activity, as evidenced in HCT116 and HeLa human cell lines (115).

The circadian rhythm of circulating cortisol in healthy adults parallels that of GR expression on leukocytes, and increases towards the end of the sleep phase (0400) and peaks shortly after waking in the morning (0800, ~400nmol/L) followed by a progressive decline towards the onset of sleep (2100, < 50nmol/L) (116). Cortisol rhythmicity is pivotal to the timing of peripheral cellular clocks to the central SCN clock (117), providing ‘time cues’ to peripheral tissues by directly increasing the expression of Per1/2 and Cry1/2. Cortisol actions on Clock and Bmal1 are indirect, occurring through its effects on these negative clock regulators. The peripheral rhythms predominantly regulated by cortisol include metabolic (glucose and lipid metabolism) and immune (suppression of inflammation during active phases) rhythms.

Total and free circulating cortisol are significantly elevated in patients with sepsis and septic shock (118–121) and higher in non-survivors than survivors (118, 121, 122). This differential may arise due to glucocorticoid resistance (123). Normally, cortisol activation of the GR inhibits CRH and ACTH release, forming a negative feedback loop. However, in GR resistance, GRα undergoes tachyphylaxis or has reduced sensitivity to cortisol due to post-translational modification, leading to a reduction in this negative feedback loop and an increase in total cortisol levels. In addition, while GRβ protein is significantly increased in mononuclear cells from humans with sepsis, and increased more than GRα in the human T-cell line CEM in response to sepsis serum (124), augmented GRβ transcription in whole blood does not correlate with *in vitro* measures of glucocorticoid sensitivity (125).

While the mesor (circadian average) of cortisol is significantly higher in patients with sepsis (62–65) reports describing characteristics of this rhythm are equivocal. A complete loss of cortisol rhythms has been reported in sepsis (62, 64, 65), while a 7-hour-delayed acrophase has also been reported (63). Hourly blood sampling has shown blunted circadian rhythms of cortisol in ICU patients with frequent cortisol pulses (126). The specific reasons for these discrepancies are uncertain, although likely arise due to the heterogeneity of patients and sampling conditions. Ultradian (<24h) cortisol secretory episodes, if large, may well obscure the circadian rhythm and are essential to map to gain a precise understanding of cortisol responses. Dysregulation of cortisol rhythms is specific to immune activation in sepsis and/or septic shock, and is unrelated to the ICU environment, as critically ill patients without sepsis exhibit normal cortisol rhythms (127). It is unknown, however, whether the circadian rhythm of GR is altered during sepsis and/or septic shock in animal models or patients.

### Melatonin

Melatonin is produced and released primarily by pinealocytes as an important sleep regulator (128) and by enterochromaffin cells in the gastrointestinal tract, where it regulates motility and inflammation (129). Melatonin binds two receptors (MtR) 1 and MtR2 that are widely distributed throughout the body; MtR1 promotes sleep, while MtR2 is involved in vasodilation and can induce phase shifts in circadian patterns. The expression of both receptors in the SCN of C3H/HeN male mice is low during the active period and increases markedly during inactive periods, a rhythm similar to that of circulating melatonin (130). In humans, melatonin levels rise in serum and urine during the night, peaking at 0000-0200, and are low throughout the day, aiding in a typical healthy sleep pattern (65, 128, 129).

Melatonin rhythms are absent in sedated and/or mechanically ventilated patients with sepsis or septic shock due to an absence of external environmental cues (64–69). However, limited reports suggest that a melatonin rhythm is present in mechanically ventilated patients but is variably shifted in acrophase to 0500 (25) or 1800 in early-stage sepsis (131), findings which may relate to discrepancies in melatonin sampling protocols. It has also been reported that a normal circadian rhythm is maintained in non-ventilated, non-sedated patients for at least the first 2 days in the ICU, after which circadian variation is lost (66, 132). However, only a subset of these patients was admitted due to sepsis, and it is not possible to separate whether sepsis or the ICU environment caused a loss of melatonin rhythm. It has also been shown that melatonin rhythms show an extended peak time during recovery from sepsis (66), and further investigation is warranted as to whether this feature follows or drives impaired recovery.

### Leptin

The adipokine leptin is a major regulator of appetite and metabolism but also plays an important role in the maturation and function of white blood cells, WBCs (133, 134). Leptin levels exhibit strong circadian rhythms in healthy adults and peak at night (~0100) with a morning nadir (~0900) (62, 135).

Leptin levels increase in patients with sepsis and murine sepsis models in association with increased pro- and anti-inflammatory cytokines, including IL-6 and IL-10 (62, 70, 136–138), while in patients, this occurs concurrent with a loss of circadian rhythm (62); whether leptin rhythmicity changes in murine models of sepsis is currently unknown. Leptin secretion from adipose tissue is increased by cortisol (139), and leptin can act as a negative regulator of the HPA axis by inhibiting CRH release (140, 141). Together, this suggests that the marked increase and loss of circadian rhythm in circulating glucocorticoids and the increase in IL-6 likely drive the increase in leptin levels in early sepsis. However, leptin levels decrease in persistent or prolonged clinical sepsis (138), likely due to a loss of fat mass. Evidence that mortality is variably increased (137) or decreased (136) in leptin receptor-deficient db/db mice subject to CLP-modelled sepsis adds to the uncertainty on whether leptin is a follower or driver of sepsis progression, and further research is required to confirm the nature of this interaction.

## Circadian machinery and the immune system in sepsis

Pathogenic infections, such as sepsis, activate Toll-like receptors on immune cells to trigger an inflammatory response mediated primarily by WBC-released cytokines. Interestingly, WBC levels in circulation show distinct circadian rhythms in healthy adults, such that hematopoietic stem cells and lymphocyte levels peak during the night (142) while neutrophil and monocyte levels peak during the day. These differential rhythms likely arise due to endocrine factors, such as cortisol, which promotes the release of neutrophils and monocytes from bone marrow (143, 144) and activates programmed cell death in lymphocytes (145).

The recruitment of immune cells to tissues during infection is mediated, in large part, via the sympathetic nervous system and predominantly occurs during the night (146), when perivascular sympathetic nerve fibres drive peak levels of WBC chemoattractant molecules from the vascular endothelium and increase endothelial cell adhesion via β-androgens and ICAM-1 (146). Immune cells are also recruited via the actions of pathogen-associated molecular patterns (i.e., Toll-like receptors, see below) and antigen presentation by dendritic cells, both of which also display circadian rhythmicity (147, 148), and are detailed below.

Whilst it is generally accepted that circulating cytokines are arrhythmic in healthy adults (149), in severe infections, including clinical sepsis, some circulating cytokines gain apparent circadian rhythmicity, such as TNFα (63). Not surprisingly, circadian rhythms of WBCs become desynchronised with phase and mesor disturbances during sepsis and septic shock, as detailed below. However, it is currently unclear whether changes in WBC levels drive or follow changes in the control of circadian machinery.

### Immune cells

#### Neutrophils

**Figure 2:**
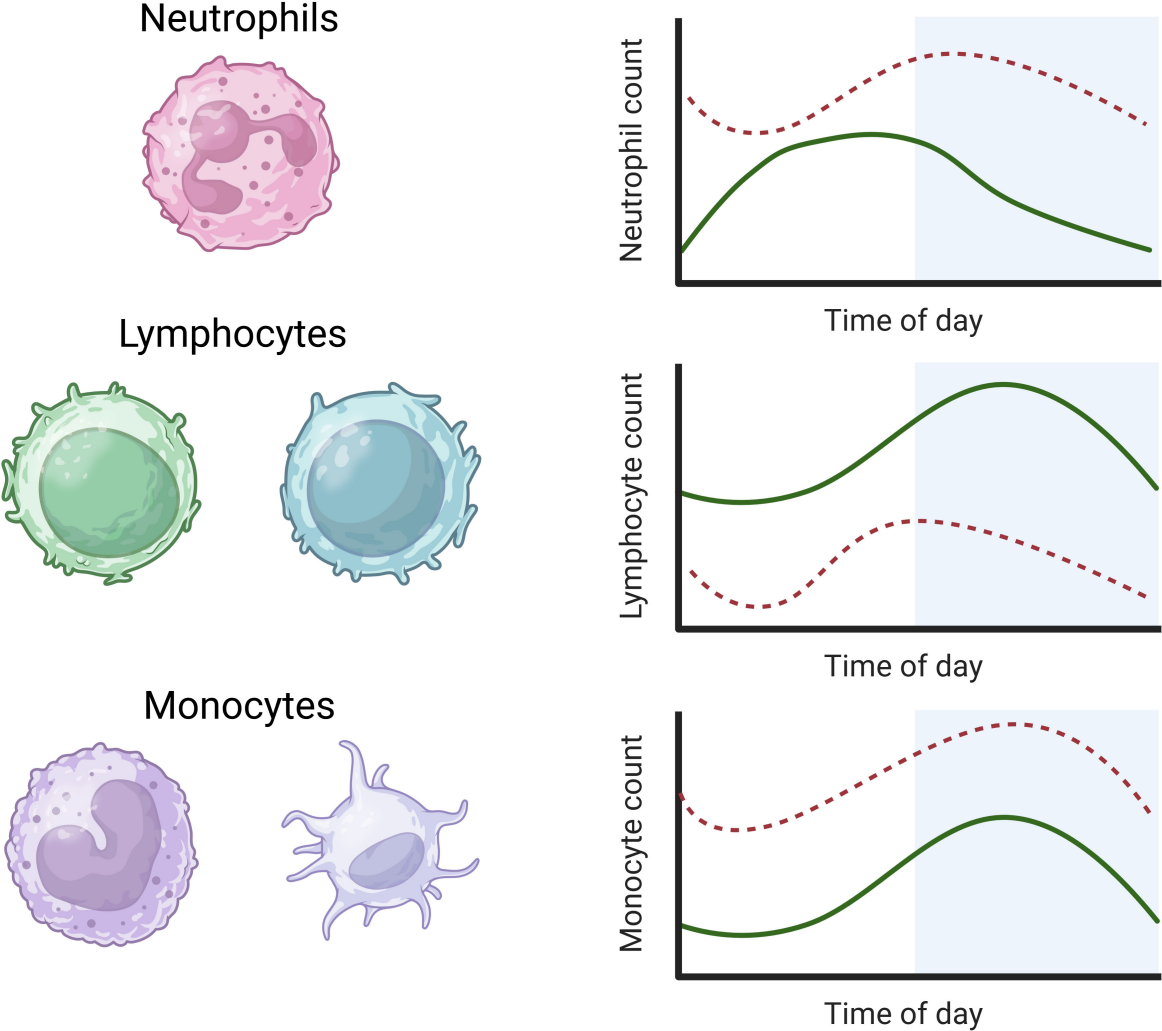
Rhythms of white blood cells in health and sepsis. Neutrophils in human plasma rhythms peak during the day, while lymphocyte and monocyte rhythms in human plasma peak during the night (solid green lines) (63, 154). In sepsis, the mesor of neutrophils is increased with a 4-hour acrophase advance. The mesor of lymphocytes is decreased in sepsis with a reverse acrophase shift of 4 hours. The circadian rhythms of monocytes show an increased mesor but no significant change in amplitude or acrophase in sepsis (dashed lines) (63) (image created in BioRender).

Circulating neutrophils constitute the majority (50-70%) WBC type in the innate immune system and recognise and destroy pathogens (150). Neutrophil markers, counts, and granules show high-amplitude circadian rhythms in the blood of healthy adults, with an extended peak during the day (1000-1400) and a nadir during the night (2200-0600) (63, 151–153). The circadian rhythm of neutrophils in human plasma during sepsis and septic shock shows an acrophase advance of +4 hours and an increased mesor, as occurs in most infections (**Figure 2**) (63).

Circulating neutrophil levels and their granulation display strong circadian rhythms in the whole blood of 7-18-week-old C57BL/6 and Balb/c male mice, in which levels peak during the day/inactive phase, in contrast to the day/active phase peak in humans (152). Neutrophils and their granulation display the same circadian rhythms in Balb/c male mice with or without neutrophil-specific Bmal1 knockout (Balb/c^Bmal1ΔN^). However, their circadian mesor is significantly decreased in the former. In contrast, these rhythms are blunted during LPS-induced sepsis in Balb/c^Bmal1ΔN^ male mice, and mortality is significantly higher (90%) than in wild-type Balb/c mice (50%) (152). This adds support that neutrophil responses to sepsis are circadian regulated and that neutrophil Bmal1 is directly protective during infection, as seen in its ability to reduce inflammation by limiting the formation of neutrophil extracellular traps which recruit immune cells and contain proteolytic enzymes that damage adjacent tissue (152). However, neutrophils and circadian clock genes also have the capacity for bidirectional control, evidenced in C57BL/6J male mice, where neutrophils that infiltrate the liver peak in a circadian manner during the day/inactive phase and indirectly increase expression of hepatic *Clock* and *Bmal1* transcription via neutrophil elastase (153).

#### Lymphocytes

B and T cell lymphocytes are key components of the adaptive immune system; B cells produce antibodies that bind foreign antigens, leading to their neutralisation or destruction, while T cells undertake a variety of immune responses, including direct killing of infected cells, activation of other immune cells, and regulation of the immune response (155, 156). Distinct from neutrophils, circulating lymphocyte counts and lymph node passage peak during the night (2200) and nadir during the day (1000) in healthy adults. Similar rhythms are observed in the plasma of C57BL/6 mice (7-8 weeks old; sex unspecified) with peaks during their inactive phase, although the passage of lymphocytes through lymph nodes shows an inverse rhythm (157). Lymphocyte levels typically rise during infection to respond to discrete pathogens. However, levels fall during a systemic inflammatory response, as in human sepsis, when the mesor is significantly reduced and acrophase reversed by −4 hours with no change in amplitude (**Figure 2**) (63). It is currently unclear what drives this acrophase shift in human sepsis, which warrants further investigation.

Based on limited evidence, it is currently unclear whether circadian rhythmicity is critical to lymphocyte functions. B and T lymphocytes are known to develop and have normal functions in C57BL/6 male mice with a lymphocyte-specific *Bmal1* knockout (158). In contrast, other studies report that a global *Bmal1* deletion reduces B lymphocyte numbers in C57BL/6 male mice (159), while C57BL/6 mice male with a global *Cry1/2* knockout show accelerated lymphocyte development and autoimmune disorders (160). This suggests that the circadian machinery within lymphocytes is not important for their development and function; rather, the clocks within the tissues that produce them, such as bone marrow, may be more important.

#### Monocytes

Monocytes are the most abundant WBC type and differentiate into macrophages or dendritic cells. Macrophages phagocytose pathogens and infected cells, while dendritic cells facilitate early responses to pathogens (161). Circulating monocyte levels display a similar rhythmicity to lymphocytes in healthy adults, peaking during the night with a nadir during the day (63, 154). This monocyte rhythm shows an increased mesor during clinical sepsis in adult humans without a change in amplitude or acrophase (**Figure 2**) (63).

A limited number of studies have investigated the circadian rhythmicity of macrophages. Two distinct macrophage populations exist within the peritoneum of C57BL/6 male mice, wherein large peritoneal macrophages are arrhythmic, while small peritoneal macrophages exhibit a circadian rhythm with a peak during the rest phase (51). LPS-induced sepsis initiated in C67BL/6 male mice during the rest phase, but not the active phase, significantly reduced the proportion of large, but not small, peritoneal macrophages (51), adding support that sepsis timing affects distinct macrophage populations.

The circadian machinery in macrophages is crucial to cytokine production. C57BL/6 male mice with a global or macrophage-specific *Clock* or *Per2* deletion release fewer cytokines, including tissue necrosis factor-α (TNFα) and interleukins, in settings of LPS- or CLP-induced sepsis (27, 55). Interestingly, as is likely with neutrophils, the circadian-macrophage relationship is bidirectional. LPS-induced sepsis increases microRNA-155 (MiR-155) in peritoneal macrophages of C57BL/6 male mice to promote haematopoiesis and the innate immune response. However, MiR-155 and *Bmal1* act directly and bidirectionally to inhibit each other in peritoneal macrophages (50). It has been further shown that LPS-induced sepsis in C57BL/6 male mice led to higher relative mortality when initiated in the rest phase when *Bmal1* is low, compared to initiation during the active phase when *Bmal1* is high (50). The authors speculated that low *Bmal1* in the early rest phase and increased MiR-155 production together exaggerated the pro-inflammatory response, which attributed to the increased mortality. In support, mortality and peritoneal macrophage MiR-155 expression increased further in *Bmal1* knockout mice with LPS-induced sepsis (50). In summary, strong lines of evidence suggest that WBCs and circadian machinery exert directional and bi-directional control during inflammation.

### Toll-like receptors

Toll-like receptors (TLRs) are crucial first responders to pathogens and trigger innate immune system activation, an effect increased by cortisol. Three heavily studied TLRs in the context of sepsis include TLR2, TRL4, and TLR9, which are present in lymphocytes and macrophages. TLR2 recognises lipoproteins and peptidoglycans from Gram-positive bacteria (162), whilst TLR4 recognises lipopolysaccharides from Gram-negative bacteria, damage-associated molecular patterns, and pathogen-associated molecular patterns (163). In contrast, TLR9 recognises bacterial and viral unmethylated CpG DNA (164). In the context of sepsis, activation of these TLRs triggers the synthesis and release of various cytokines from immune cells, including TNFα, IL-6, and IL-12, driving the excessive inflammatory response.

#### TLR2

TLR2 is present on cell plasma membranes and intracellular endosomes to respond to bacteria that have been internalised and is involved in the production of cytokines in response to sepsis. TLR2 transcription is arrhythmic in splenocytes of healthy 8-week-old C57BL/6 male mice, but rhythmicity was reported in splenic-derived macrophages that peaked during the active phase at ZT-19, highlighting cell-specific TLR2 regulation (165). CLP in C57BL/6 mice (sex unspecified) was shown to trigger an increase in splenic mRNA content of IL-6, IL-10, and MIP-2 within 6 hours, an effect that was lost in TLR2^-/-^ mice, who had similar mRNA levels to sham-operated mice (166), although it was unclear whether this change occurred in splenocytes, WBCs or both. Blockade of TLR2 with a monoclonal T2.5 anti-mouse antibody, prior to or 3 hours after CLP in 8-12-week-old C57BL/6 male mice, was shown to reduce the mortality rate to 25% and 60%, respectively, compared to non-treated controls (100% mortality within 48 hours) (167). This finding adds support to the potential for TLR2 blockade as a sepsis therapeutic. Interestingly, the difference in mortality related to light or dark phase induction of CLP was lost in TLR2^-/-^ female mice (52), indicating that TLR2 may play a role in circadian-dependent mortality outcomes in murine models of sepsis.

#### TLR4

TLR4 is also present in plasma cell membranes and endosomes. In contrast to TLR2, adherent splenocytes from healthy C57BL/6 male mice show a rhythm of TLR4 transcription that peaks at ZT-14 in the early active phase, while splenic-derived macrophages are arrhythmic, to highlight splenic cell-specific TLR2 and TLR4 rhythmicity (55). C57BL/6 male mice deplete in TLR4, or C3G/HeJ male mice with a missense point mutation that lacks TRL4 only in the cytoplasm, both show reduced sensitivity to LPS, suggesting that TLR4 is required for infection responses (168). While the potential for circadian rhythm change in TLR4 production during modelled sepsis is unmapped, the TLR4 increase during the active phase may be integral to circadian-dependent effects of mortality in murine models of sepsis.

#### TLR9

TRL9 is mainly located within intracellular endosomes and lysosomes, where it recognises pathogenic DNA internalised by immune dendritic and B cells. The TLR9 promotor contains *Clock* and *Bmal1* binding motifs (55), while TLR9 expression in C57BL/6 B lymphocytes shows circadian rhythmicity with peak expression at ZT16 during the active phase (55). CLP performed in C57BL/6 mice at this TLR9 peak shortened the time to 100% mortality compared to CLP performed in the inactive phase, which the authors attributed to this TLR9 rhythm peak (55). Global depletion of TLR9 in Balb/c mice (TLR9^-/-^) led to significantly lower CLP mortality compared to wild-type controls (25 vs. 75%, sex and CLP time unspecified) and lower circulating levels of IL-6, IL-10, and TNFα (169). It is also known that C57BL/6 TLR9^−/-^ mice show lower CLP mortality compared to wild-type mice (20 vs. 100%, sex and CLP time unspecified) (170). However, evidence that CLP performed in the active phase (ZT19) led to higher mortality compared to the inactive phase (ZT7) in 6-12-week-old C57BL/6 TLR9^−/-^ mice (80 vs. 30%, mixed sex) suggests that TLR9 is not involved in the circadian dependency of sepsis mortality *per se* (52). Nonetheless, despite no association with a greater risk of death, evidence that a gain of function polymorphism in TLR9 (TLR9-1237T/C) is associated with a two-fold increased risk of severe sepsis and progression to shock in paediatric patients (median age 8 months) has piqued interest in TLR9 as a future target for sepsis treatment (171).

### Cytokines

#### TNFα

TNFα is released primarily from monocytes such as macrophages and binds its cognate receptor TNF-Receptor (TNFR) 1 with higher affinity than TNFR2. TNFR1 activation then activates the Nuclear Factor-κB (NF-κB) or Death Inducing Signalling Complex (DISC); NF-κB plays a role in the activation of inflammation (172) whilst DISC contains procaspase 8, which triggers a signalling cascade that causes cell death (173). Serum and tissue levels of TNFα and TNFR1 are known to be markedly increased during sepsis in humans, rats, and mice (25, 26, 55, 63, 174), while it has been shown that *Clock* deletion reduces macrophage-derived TNFα (27).

Serum and tissue TNFα levels do not exhibit circadian rhythmicity in healthy adults and rodents (25, 55, 63, 174). However, TNFα levels increase markedly in the setting of clinical sepsis and acquire a circadian rhythm that peaks at night in human serum (25, 63) and during the rest phase in rat hippocampal microglia (26). LPS-induced sepsis in C57BL/6 male mice timed to the rest phase has also been shown to align with higher serum TNFα concentrations compared to sepsis initiation during the active phase (51). However, other studies have shown no difference in the magnitude of TNFα increase in serum or the whole CNS pre-optic area in response to LPS in C57BL/6 male mice aligned to circadian rhythms (49, 51). This highlights the need for additional research, as well as the potential for sepsis-dependent TNFα increases to exhibit tissue or compartment-specific circadian rhythms.

#### Interleukins

Interleukins are produced by many WBCs and are involved in the differentiation and activation of other immune cells, with IL-1β, IL-6, and IL-10 being the most studied in the context of sepsis (25, 63). IL-1β inhibits cell proliferation, promotes apoptosis, and leads to the activation of cyclooxygenase 2, a pro-inflammatory enzyme, in all tissues in humans (175). IL-6 triggers the production of various inflammatory markers, including hepatic C-reactive protein and serum amyloid A in humans, both of which are involved in cytokine production and recruitment of immune cells (176), respectively, while IL-10 attenuates the release of cytokines from human T cells but also aids B cell proliferation and survival (177).

There is general agreement that interleukins are arrhythmic in human health and sepsis. However, as for TNFα, the magnitude of the rise in circulating interleukin due to LPS sepsis shows circadian dependency. For example, IL-1β and IL-6 concentrations are higher in the hippocampus and serum of Sprague-Dawley male rats and C57BL/6 male mice with LPS-induced sepsis initiated during the dark compared to light phase (26, 49). In contrast, while sepsis increases serum IL-10, the magnitude of this increase is not circadian-dependent (26, 49, 51), and this distinct control of pro- and anti-inflammatory cytokines warrants further investigation.

### Body temperature

The body temperature of humans and most mammals follows a circadian rhythm with a sinusoidal fluctuation of about 0.5-1°C, peaking approximately 12 hours following the onset of daylight with a nadir during the night (178). Body temperature increases during the acute phase of infection to enhance the efficacy of WBCs and to induce pathogenic stress. This is observed in early-stage sepsis as body temperature rhythm changes to one with increased mesor, amplitude, and frequency (179), in essence, shifting to an ultradian rhythm (rhythm with several cycles of at least 1 hour throughout 24 hours). Patients with decompensated sepsis and high sequential organ failure scores often also exhibit hypothermia, which is linked to a significant increase in mortality (180, 181). It has been suggested that hypothermia during sepsis parallels the body entering an ‘energy saving’ mode, compared to hyperthermia in ‘active infection-fighting’ mode (182). LPS-induced sepsis in C57BL/6 male mice produced a larger and more rapid drop in body temperature in the light- compared to the dark phase (51), which was postulated due to hypothalamic overactivation during the light phase (measured as cFOS expression) (51). Overall, the effects of sepsis on the circadian rhythms of body temperature are not well elucidated, and substantial research is required for full knowledge.

## Implications for chronotherapy

Chronotherapy leverages circadian rhythms to augment or improve medical treatments over two main avenues. The first is to restore a regular sleep/wake cycle in specific pathologies where circadian disruption exacerbates disease. The second is to time administration of medication to existing circadian rhythms, also known as chronopharmacology (183).

### Chronotherapy

Chronotherapy has been used to aid the treatment of mood and depressive disorders. For example, bright light therapy is used to treat seasonal and non-seasonal affective disorders, such as seasonal depression and major depressive disorder, with significant improvements noted in self-reported depression (184, 185). Other studies have used a combination of sleep deprivation for 24–48 hours and bright light therapy for 1 week to evidence a rapid and sustained reduction in bipolar and depressive symptoms (186, 187). In addition, using urinary melatonin as a marker of circadian rhythms, it was shown that exposure to a 400-700 lux light box for 3 hours in the morning (0900-1200) re-aligned circadian rhythms within 48 hours in phase-delayed ICU patients (188). Together, these studies highlight light and/or sleep cycle manipulations in current use to re-align circadian rhythms and effectively treat disorders, notably for improved mental health.

### Chronopharmacotherapy

Chronopharmacotherapy leverages circadian rhythmicity to optimise management of several clinical conditions. For example, administration of H2 antagonists to treat duodenal ulcers is optimal at bedtime when gastric acid secretions are highest (189). Administration of non-steroidal anti-inflammatory drugs is optimal in the afternoon for arthritis and at night for rheumatic disorders (190, 191). Cortisol replacement therapy also leverages chronobiology in administering delayed-release cortisol once daily at night (i.e., Plenadren) or twice daily (i.e., Chroncort), with improved well-being reported in patients with adrenal insufficiency (192). In addition, inflammatory disorders such as rheumatoid arthritis and ankylosing spondylitis are optimally treated by early morning glucocorticoid therapy to manage peak symptom severity in the late evening and early morning in humans when pro-inflammatory cytokines peak (193). Together, these highlight the value of chronopharmacotherapy within current practice, and potential benefits on offer to leverage circadian machinery and rhythms to optimise disease treatment in the future.

Few studies have investigated the use of chronotherapy to treat immune responses in sepsis. Twenty-four hours of above-cage blue light (442 nm, lux 1400) exposure after CLP in C57BL/6 male mice increased splenic Rev-Erb protein and reduced peritoneal bacterial colony formation as well as serum TNFα, IL-6, and IL-12, compared to ambient light. This effect was lost in C57BL/6 male mice deficient in Rev-Erb, and likely relates to Rev-erb actions to suppress cytokine production (194), a notion that garners support in evidence that Rev-Erb agonists suppress cytokine production by LPS-treated human alveolar macrophages (195). Benefits of this same blue-light therapy over 18 hours were also shown in patients with appendicitis, who had lower postoperative serum IL-6 levels relative to patients under normal light (194). Exposure to blue light (442 nm, lux 1400) for 24 or 36 hours, followed by 12 hours per day for 72 hours in C57BL/6 male mice with a monobacterial *Klebsiella pneumoniae* infection led to significant reductions in mortality (40%) compared to mortality under ambient (75%) or red light (80%) (196). Together, these blue light intervention studies frame the potential for blue light chronotherapy to treat or augment the efficacy of other sepsis therapies and warrant deeper investigation.

Melatonin has been tested in the context of sepsis therapy in infants, where oral melatonin (10 mg twice for one day) significantly improved recovery time and reduced WBC count (197). In a more recent study, oral melatonin (50 mg daily for five days) was shown to significantly reduce sequential organ failure score and decrease vasodilator and ventilator dependency in adults with sepsis (198). While promising, further research is needed to elucidate whether melatonin effects arise due to circadian or anti-inflammatory actions or both.

## Conclusions

Ample lines of research evidence the integral role circadian rhythms play in normal physiological functions and their potential, in disruption, to progress disease. Despite the challenges of blunted or phase shifted rhythms in sepsis and septic shock, and the need to define whether these drive or follow disease progression, the utility of chronotherapy evident in preclinical sepsis research, and proven clinical efficacy emerging in mood disorders and appendicitis (blue light therapy), stands to benefit sepsis and septic shock management. Multidisciplinary collaborations are now required to translate preclinical findings on sepsis chronotherapy to clinical practice, to restore circadian rhythms and align treatment in a manner that personalises and improve outcomes.
